# Supplementary material for: Congenital Pseudarthrosis of the Clavicle in Children: A Systematic Review
Source: Children (Basel). 2022 Jan 24;9(2):147. doi: 10.3390/children9020147 (PMC8870275; doi:10.3390/children9020147)
Supplement: Supplementary file 1 [file children-09-00147-s001.zip › children-1533991-supplementary.pdf]

SUPPLEMENTARY MATERIALS

Table S1. results from screening of case reports in English Literature with less than three cases.

**Patients with CPC**	53
With thoracic outlet syndrome (TOS)	4 patients > 18 y, 1 patient aged 15 y
**Children with CPC**	46 (56 clavicles)
Sex	24 males, 20 females, 2 unspecified
Side	34 right, 2 left, 10 bilateral
Treatment	27 conservative, 19 surgery
Complications of surgery	1 minor, 6 major

Figure S1: graph showing logistic regression between symptoms at presentation and age (β-coefficient 0.116, $p = 0.093$).

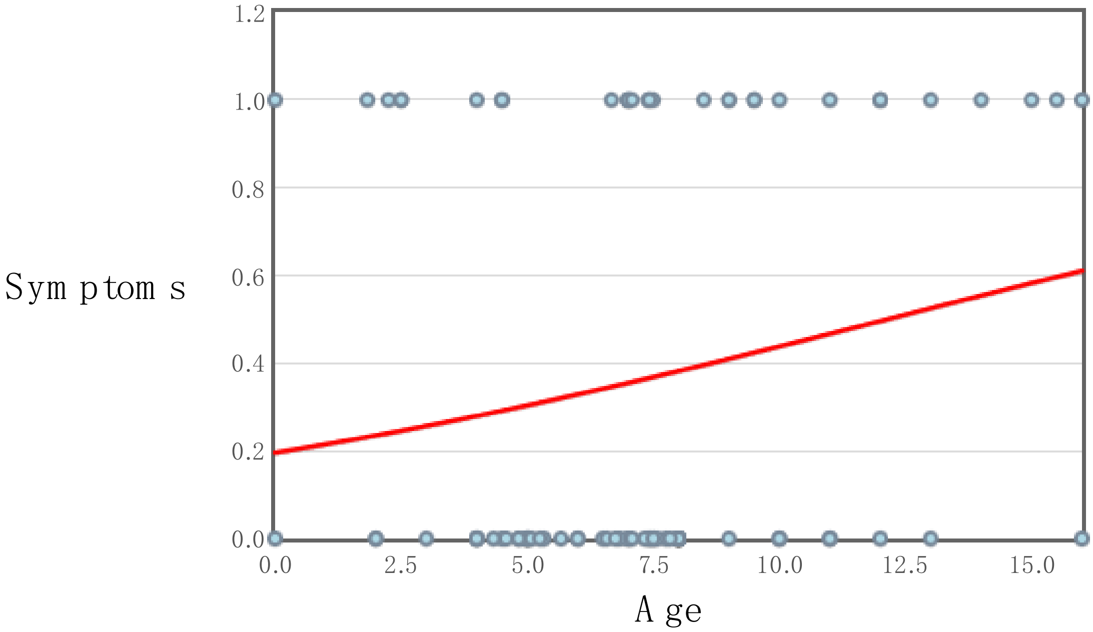

Figure S2: graph showing logistic regression between healing rate (union = 1, non-union = 0) and age at treatment (β-coefficient = -0.124, $p = 0.156$).

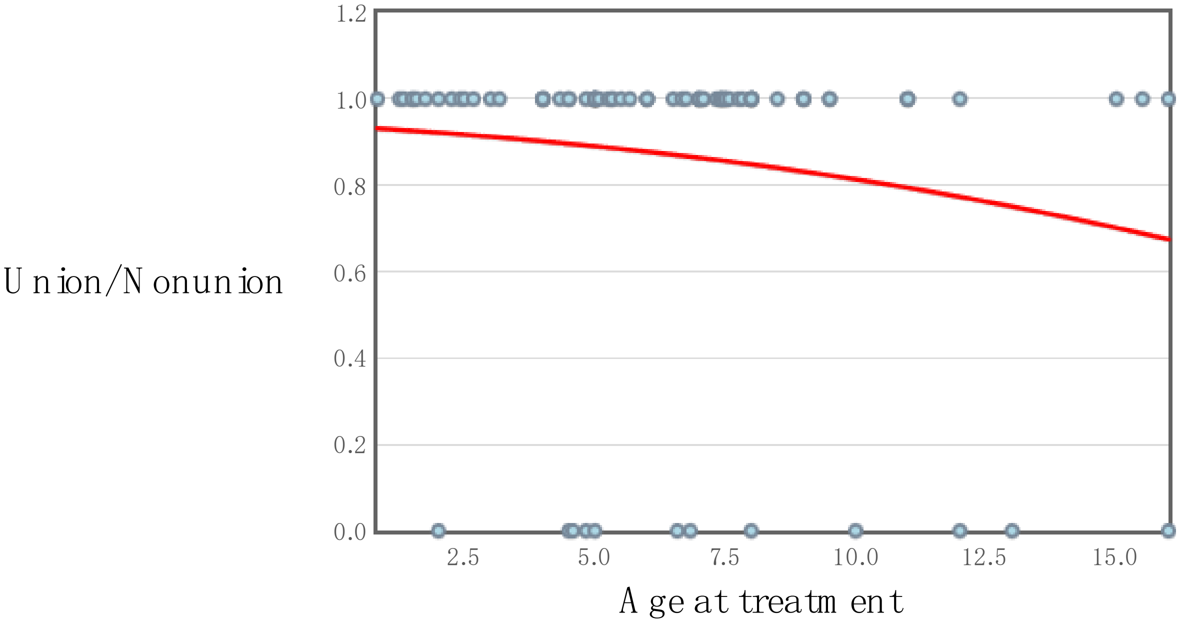

Table S2: comparison of non-union rate between surgical groups. Excision was not included since non-union was observed in all 5 cases. The Fisher's exact test (yellow background) and the Chi-squared test (green background) showed no significant difference between surgical groups.

Graft	Graft + BS	Graft + pin	Graft + plate	Masquelet	
0.549	1.000	0.625	0.296	1.000	**Fixation**
	0.601	1.000	1.000	1.000	**Graft**
		0.455	0.361	1.000	**Graft + BS**
			0.278	1.000	**Graft + pin**
p-value nonunion rate				1.000	**Graft + plate**

Figure S3: **(a)** the logistic regression found no correlation between age at treatment and complications (complication = 1, normal = 0; β-coefficient = -0.044, p = 0.574); **(b)** linear regression between age at treatment and Clavien-Dindo-Sink (CDS) score found no correlation at all (r = 0.023, p = 0.830).

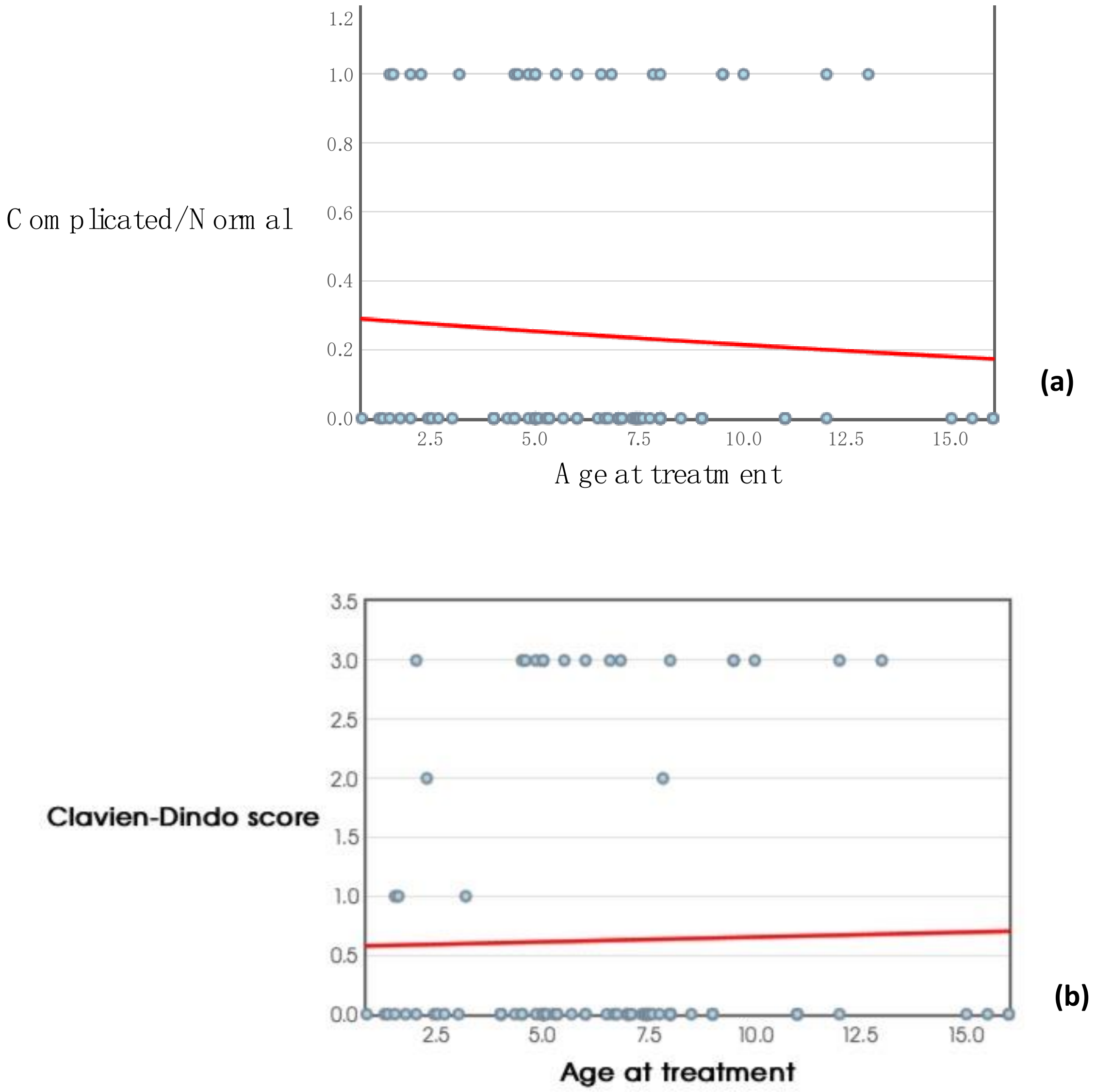

Table S3: comparison complication rate between surgical groups; none of the surgical group had a significantly higher prevalence of complications The Fisher's exact test (yellow background) and the Chi-squared test (green background) showed no significant difference between surgical groups.

Fixation	Graft	Graft + BS	Graft + pin	Graft + plate	Masquelet	
0.505	1.000	0.530	0.313	1.000	0.107	**Excision alone**
	0.615	1.000	1.000	0.646	0.236	**Fixation**
		0.390	0.333	1.000	0.442	**Graft**
			1.000	0.440	0.272	**Graft + BS**
p-value complication rate				0.093	0.231	**Graft + pin**
					0.091	**Graft + plate**
